# A new genus of minute Sparianthinae spiders from the Caribbean (Araneae, Sparassidae)

**DOI:** 10.3897/zookeys.742.21819

**Published:** 2018-03-12

**Authors:** Cristina Anne Rheims, Giraldo Alayón

**Affiliations:** 1 Laboratório Especial de Coleções Zoológicas, Instituto Butantan, Av. Vital Brasil, 1500, 05503-900, São Paulo, SP, Brasil; 2 Museo Nacional de Historia Natural, Obispo No. 61, Ciudad de La Habana, C.P. 10100, Cuba

**Keywords:** Cuba, *Diminutella* gen. n., taxonomy, West Indies

## Abstract

The monotypic genus *Diminutella* **gen. n.** is proposed to include the type species, *Diminutella cortina* **sp. n.**, described from San Diego de los Baños, based on male and female specimens. This species is amongst the smallest Sparianthinae known to date reaching only 4.4 mm body length. It can be distinguished from other Sparianthinae by the combination of eye arrangement, with anterior eye row recurved, posterior eye row straight, spination pattern of legs I–II, with three ventral pairs on tibiae and one on metatarsi, and by the peculiar genitalia, with male palps bearing a large, laminar, layered conductor that rests within a retrolateral depression in the cymbium, and a female vulva with long copulatory ducts coiled around the stalk of a globose blind ending appendage.

## Introduction

Sparianthinae Simon, 1897 is a well-established subfamily of Sparassidae Bertkau, 1872, characterized by the presence of a trilobate membrane with small or inconspicuous median lobe on metatarsi I–IV, small retromarginal denticles on the chelicerae and a median apophysis on the male palps (Jäger 1998, Ramírez 2014). To date, it includes 115 species distributed in 16 genera: *Decaphora* Franganillo, 1931, *Defectrix* Petrunkevitch, 1925, *Neostasina* Rheims & Alayón, 2016, *Pleorotus* Simon, 1898, *Pseudosparianthis* Simon, 1887, *Rhacocnemis* Simon, 1897, *Sagellula* Strand, 1942, *Sampaiosia* Mello-Leitão, 1930, *Sparianthis* Simon, 1880, *Stasina* Simon, 1877, *Stipax* Simon, 1898, *Strandiellum* Kolosváry, 1934, *Stasinoides* Berland, 1922, *Thelcticopis* Karsch, 1884, *Thomasettia* Hirst, 1911 and *Uaiuara* Rheims, 2013 (World Spider Catalog 2018). Nevertheless, the placement of *Stasinoides* Berland, 1922 and *Sagellula* Strand, 1942 (with one and two species, respectively) within the subfamily, or even the family, is considered doubtful (Jäger and Yin 2001, Jäger and Kunz 2005).

The subfamily occurs worldwide, but the Neotropical region is particularly rich in terms of Sparianthinae fauna and recent papers (Rheims 2013, 2017, Rheims and Alayón 2014, 2016) show that the number of new species and genera is growing as its systematics is studied. Eight genera and 54 species are known from the region: *Decaphora* (5 species), *Defectrix* (1 species), *Neostasina* (27 species), *Pseudosparianthis* (8 species), *Sampaiosia* (1 species), *Sparianthis* (1 species) *Stasina* (4 species) and *Uaiuara* (7 species) (World Spider Catalog 2018). The four Neotropical species currently included in *Stasina* are not congeneric with the type species, *Stasina spinosa* Simon, 1897, and are considered misplaced in the genus (Rheims and Alayón 2016). Most are only known from juveniles and cannot be placed in any other genera until the adults are collected. The monotypic genus *Defectrix* is known solely from a juvenile. However, the original description includes traits that are not found in Sparianthinae, such as notched trochanters (smooth in Sparianthinae), only two pairs of ventral spines on tibiae I–II (three or more in Sparianthinae) and absence of a colulus (present in Sparianthinae) (Petrunkevitch 1925). We consider it misplaced in the subfamily and thus, consider that only six genera are known from the Neotropical region.

In this paper, we describe the new genus *Diminutella* gen. n. to include a species of minute Sparianthinae, *Diminutella cortina* sp. n., found in San Diego de los Baños, western Cuba. It is one of the smallest species of Sparianthinae known to date, with total body lengths ranging from 3.5 to 4.4 mm. Amongst Neotropical Sparianthinae, similar sizes are only found in the genus *Uaiuara*, with total lengths ranging from 3.7–10.0 mm (Rheims 2013). Other small, but slightly larger species belong to the Afrotropical genera *Stipax* Simon, 1898 and *Pleorotus* Simon, 1898, both monotypic and endemic to the Seychelles Islands (Simon 1898).

In the absence of a cladistic analysis, not much can be said about the relationships between *Diminutella* gen. n. and the remaining Sparianthinae genera. However, despite being known from a single species, we consider that the genus represents a monophyletic unit. *Diminutella* gen. n. shows a combination of somatic characters that is not found in any other genera of Neotropical Sparianthinae and both male and female genitalia are markedly different from all other species known to date. The importance of spines, male palp and female epigyne features was previously pointed out by Rheims & Alayón (2016). Also, Sparassidae species are usually conservative and rarely deviate from the generic *bauplan*. We consider that, if new species of *Diminutella* gen. n. are discovered in the future they will show the same characters that are currently used to diagnose the genus and will strengthen our hypothesis that this is a separate lineage within Sparianthinae.

## Materials and methods

The examined material is deposited in the collections of the Museo Nacional de Historia Natural de Cuba, La Habana, Cuba (**MNHNCu**, curator: G. Alayon) and National Museum of Natural History, Smithsonian Institution, Smithsonian Institution, Washington D.C., USA (**NMNH**, curator: J.A. Coddington).

Morphological observations and illustrations were made using a Leica MZ165C stereomicroscope with a camera lucida. Measurements were taken with a micrometric ocular and are given in millimeters. Female genitalia were examined in clove oil after dissection. Photos of dorsal habitus, male palps, and female epigyne were taken using a Leica DMC 4500 camera attached to a Leica M205 A stereomicroscope. Extended focal range images were composed with the program Leica Application Suite version 4.8.0. SEM images were taken with a FEI Quanta 250 scanning electron microscope from the Laboratório de Biologia Celular at the Instituto Butantan. Material used for SEM was mounted on metal stubs and sputter coated with gold.

Abbreviations used throughout the text are given bellow.

**Somatic morphology**

**AER** anterior eye row

**ALE** anterior lateral eyes

**ALS** anterior lateral spinnerets

**AME** anterior median eyes

**d** dorsal

**p** prolateral

**PER** posterior eye row

**PLE** posterior lateral eyes

**PLS** posterior lateral spinnerets

**PME** posterior median eyes

**PMS** posterior median spinnerets

**r** retrolateral

**v** ventral

**Male palp**

**dRTA** dorsal branch of RTA

**RTA** retrolateral tibial apophysis

**vRTA** ventral branch of RTA

**VTA** ventral tibial apophysis

Format of descriptions follows Rheims (2007). Spine notation follows Petrunkevitch (1925). Leg measurements are listed as: total length (femur, patella, tibia, metatarsus, tarsus); eye diameters as: AME, ALE, PME, PLE; and interdistances as: AME–AME, AME–ALE, PME–PME, PME–PLE, AME–PME, ALE–PLE. Positions of tegular appendages are given according to clock positions, based on the left male palp in ventral view. In schematic illustrations of female vulva the blind ending appendage is marked with “T”, the copulatory opening with a circle, and the end of the fertilization duct in direction of the uterus externus with an arrow. Coloration patterns are described based on specimens preserved in 70% ethanol. Only characters that differ from those of the male are mentioned in the female description. Geographical coordinates of the collection locality were obtained from Google Earth.

## Taxonomy

### Family Sparassidae Bertkau, 1872

#### Genus Diminutella gen. n.

Taxon classificationAnimaliaAraneaeSparassidae

http://zoobank.org/2AFEC3A5-8B40-483E-BD13-CFFFE13E0351

##### Type species.

*Diminutella cortina* sp. n.

##### Etymology.

The specific name is a combination of the Brazilian adjective “diminuto” and the suffix “ella” and refers to the minute size of the specimens of the type species of the genus, considered the smallest Sparassidae known to date. Gender is feminine.

##### Diagnosis.

**Figure 1.**
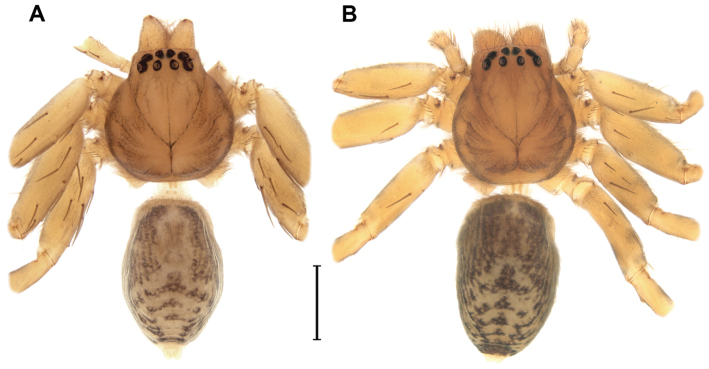
*Diminutella cortina* sp. n., habitus, dorsal view A male (paratype) B female (paratype). Scale lines: 1mm.

**Figure 2.**
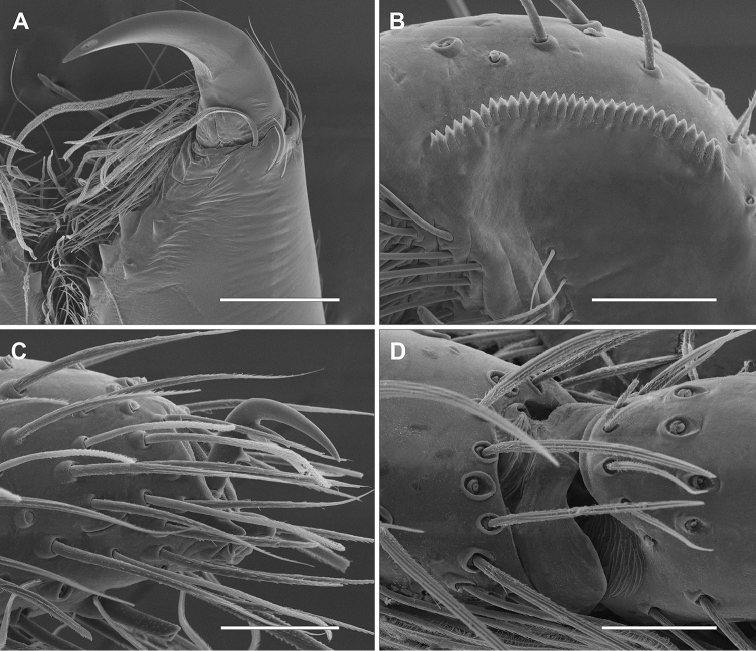
*Diminutella cortina* **sp. n.** (female). **A** left chelicerae, ventral **B** left endite, serrula, ventral **C** right palp, prolateral **D** metatarsus IV, dorsal. Scale lines: 400μm (**A**), 50μm (**B–D**).

Species of *Diminutella* gen. n. can be distinguished from all known Neotropical genera by the combination of the following characters: AER recurved and PER straight (Figs 1A–B), chelicerae with three promarginal teeth and two retromarginal denticles (Fig. 2A) and legs I–II with three ventral pairs of spines on tibiae and one on metatarsi. They are distinguished from *Decaphora* by the eye arrangement and cheliceral dentition (AER straight, PER procurved, four promarginal teeth and 5–7 retromarginal denticles in *Decaphora*); from *Neostasina* by the eye arrangement (AER straight and PER procurved in *Neostasina*); from *Pseudosparianthis*, *Sampaiosia* and *Sparianthis* by the number of spines on metatarsi I–II (two pairs in these three genera) and by the eye arrangement (AER and PER procurved in *Pseudosparianthis* and *Sampaiosia* and AER and PER recurved in *Sparianthis*); and from *Uaiuara* by the number of ventral spines on tibiae I–II (7–9 pairs in *Uaiuara*).

##### Description.

See species description.

##### Species composition.

Monotypic: *Minutella cortina* sp. n.

##### Distribution.

Pinar del Rio, Cuba.

#### Diminutella cortina sp. n.

Taxon classificationAnimaliaAraneaeSparassidae

http://zoobank.org/EAF7509E-996F-45D6-A4AA-A11FC254F878

**Figure 3.**
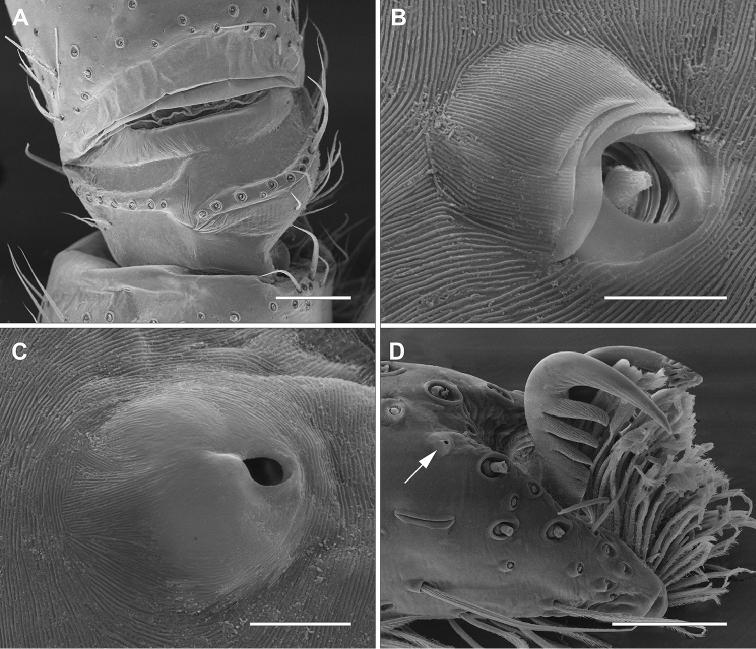
*Diminutella cortina* **sp. n.** (female). **A** trocanther I, ventral **B** tarsus III, tricobothria, dorsal **C** tarsus IV, tarsal organ, dorsal **D** tarsus IV, prolateral (arrow pointing at tarsal organ). Scale lines: 100μm (**A**), 5μm (**B–C**), 50μm (**D**).

**Figure 4.**
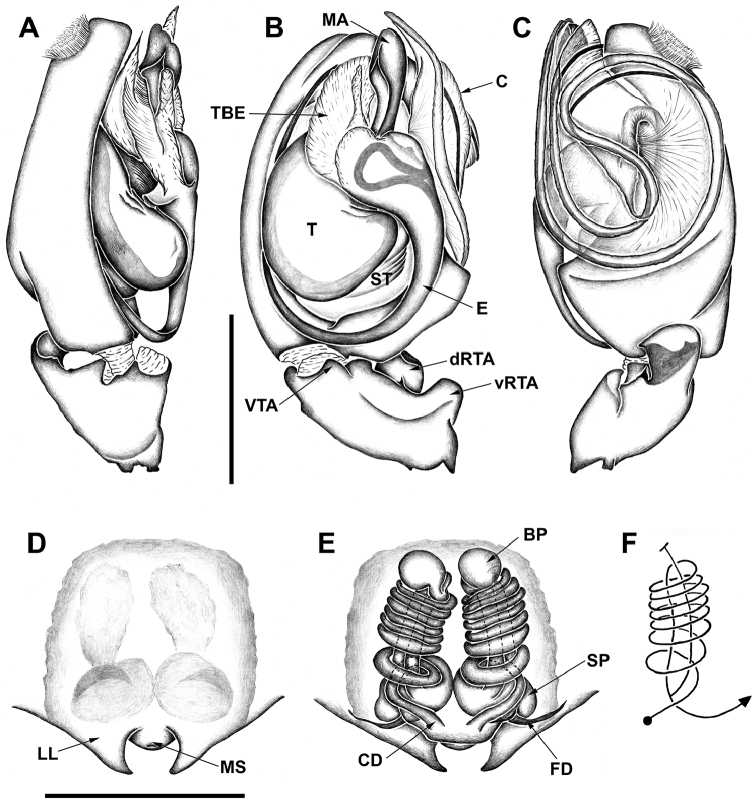
*Diminutella cortina* **sp. n. A–C** male (holotype), left palp **A** prolateral **B** ventral **C** retrolateral **D–F** female (paratype) **D** epigyne, ventral **E** vulva, dorsal **F** schematic course of internal duct system. BP – blind ending appendage; C – conductor; CD – copulatory duct; dRTA – dorsal branch of RTA; E – embolus; FD – fertilization duct; LL – lateral lobe; MA – median apophysis; MS – median septum; SP – spermathecae; ST – subtegulum; T – tegulum; TBE – tegular projection at base of embolus; vRTA – ventral branch of RTA; VTA – ventral tibial apophysis. Scale lines: 0.5 mm.

**Figure 5.**
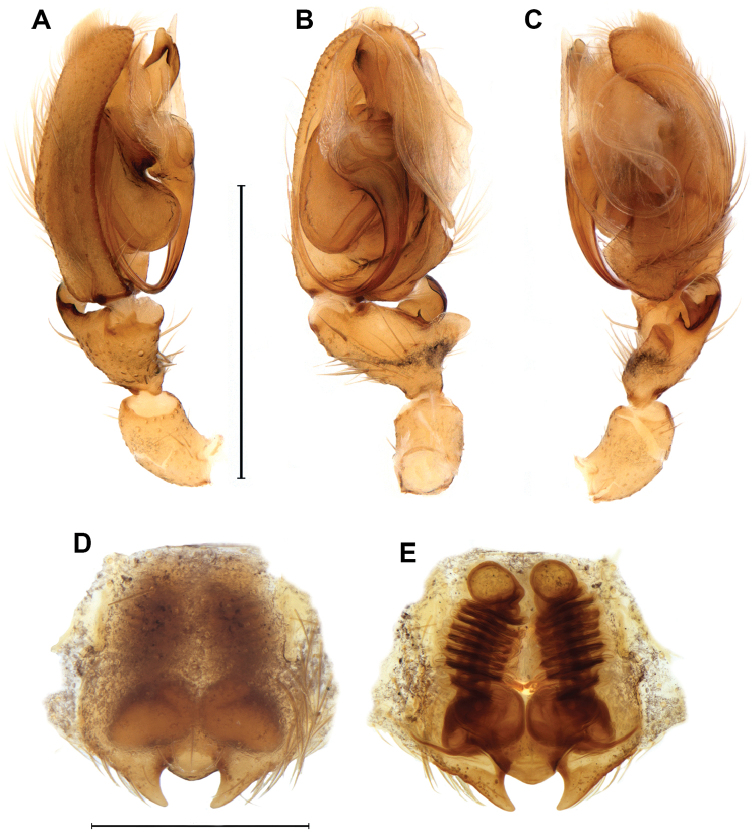
*Diminutella cortina* sp. n. **A–C** male (holotype), left palp **A** prolateral **B** ventral **C** retrolateral **D–E** female (paratype) **D** epigyne, ventral **E** vulva, dorsal. Scale lines 1mm (**A–C**), 0.5mm (**D–E**).

Figs 1 , 2 , 3 , 4 , 5

##### Types.

Holotype ♂: CUBA, Pinar del Rio, San Diego de los Baños [22°38'N, 83°22'W], Hacienda Cortina, September 1985, G. Alayón leg. (NMNH 4637A). Paratypes: 1♀ (NMNH 5570A), 1♂ and 2♀, (MNHNCu), same collection data as holotype.

##### Etymology.

The specific name refers to the type locality of the species, Hacienda Cortina.

##### Diagnosis.

*Diminutella cortina* sp. n. is distinguished from those of the remaining Neotropical Sparianthinae genera by the male palp with large hyaline, layered, laminar conductor resting within retrolateral cymbial depression (Figs 4C, 5C) and by the female vulva with long copulatory ducts arising from posteriorly located openings, coiling around the duct leading to the blind ending projection and the part of the copulatory duct leading to the posteriorly situated spermathecae (Figs 4E–F, 5E).

##### Description.

*Male (holotype)*. Prosoma orange with pale brown margins, thin dark brown line along lateral margins of cephalic region and fovea and faint brown reticulated pattern extending posteriorly from behind posterior eyes to thoracic region. Eye borders black (Fig. 1A). Chelicerae, legs and palps orange. Labium and endites pale yellow with pale orange base. Sternum pale yellow with slightly darker margins. Opisthosoma cream colored. Dorsally with brown pattern of longish marks laterally and around cardiac mark and posterior median chevron-like marks down posterior half (Fig. 1A). Ventrally with faint brown, elongated marks laterally and few scattered brown spots. Spinnerets yellowish cream colored. Total length 4.4. Prosoma: 2.0 long, 1.8 wide; cephalic region slightly higher than thoracic region, gradually flattening posteriorly; fovea conspicuous on posterior third of prosoma. Eyes: eight eyes arranged in two rows, the anterior slightly recurved, the posterior straight; AME as large as ALE and equidistant; PME smaller than PLE and equidistant (Figs 1A–B); diameters: 0.12, 0.12, 0.10, 0.13; interdistances: 0.08, 0.08, 0.16, 0.16, 0.09, 0.06. Clypeus low, less than AME diameter. Chelicerae with three promarginal teeth, the basal one smallest, and two retromarginal denticles; intermarginal denticles absent; one single strong setae at the base of fang (Fig. 2A). Labium slightly longer than wide. Endites converging anteriorly, with dense scopulae on internal margin. Serrula with a single row of denticles (Fig. 2B). Sternum as long as wide, slightly projected between coxae IV. Legs laterigrade, 4213; trochanter smooth (Fig. 3A); metatarsi I–IV distally with dorsal trilobate membrane with median hook much smaller than lateral projections (Fig. 2D); tarsi and distal half of metatarsi scopulate; trichobothria present on dorsal tibiae, metatarsi and tarsi, arranged in several rows that converge to a single row at the posterior end of tarsi and metatarsi; bothrium with crescent plate, with 2–3 grooves, projected over a smooth basal plate (Fig. 3B); tarsal organ capsulate with oval opening (Fig. 3C), located dorsally at the distal end of tarsi (Fig. 3D); tarsi with claw tufts and a pair of pectinate claws with four very slightly curved teeth (Fig. 3D); measurements: I: 5.9 (1.7, 0.8, 1.5, 1.4, 0.5); II: 6.6 (1.9, 1.0, 1.7, 1.5, 0.5); III: 5.2 (1.7, 0.7, 1.0, 1.4, 0.4); IV: 6.8 (1.9, 0.8, 1.7, 1.8, 0.6); spination: femora I–III: p1-1-1, d0-1-1, r1-1-1; femur IV: p1-1-1; d0-1-1; r0-0-1; tibiae I–IV p1-0-1; d0-0-1; r1-0-1; v2-2-2; metatarsi I–II: p1-1-0; r1-1-0; v2-0-0; metatarsus III: p1-2-1; r1-1-1; v2-2-0; metatarsus IV: p1-1-1, r1-1-1, v2-2-0. Metatarsus III–IV with ventral distal preening combs. Opisthosoma: 2.3 long, 1.4 wide. Six spinnerets: ALS contiguous, conical and bi-segmented. Basal segment elongate and cylindrical, distal segment short and truncated. PMS conical and short. PLS conical and bi-segmented. Basal segment elongate and cylindrical, distal segment short and truncated. Palp: tibia slightly longer than patella with one prolateral spine proximally; VTA present, situated medially; RTA with vRTA rectangular in ventral view, distally laminar and dRTA spoon-shaped with small prong-like projection at base; cymbium rounded with subdistal dorsal scopula; subtegulum visible retrolaterally in ventral view, with 5-6 anneli; tegulum C-shaped; embolus long, filiform with large bulbous base, arising from tegulum at 1:30 o’clock position; tegular projection at base of embolus hyaline, with two lobes, the posterior fan-like and laminar and the anterior conical, arising from the bulbous base of the embolus; median apophysis cup-shaped, with margin strongly indented medially, arising from tegulum at 1 o’clock position (Figs 4A–C, 5A–C); spination: femur: p0-0-1; d0-1-2; r0-0-1; tibia p0-1.

*Female (Paratype, NMNH 5570A).* Coloration pattern generally darker than in male. Prosoma brown with dark brown pattern as in male (Fig. 1B). Chelicerae brown. Legs and palps pale brown labium and endites pale orange, distally translucent white. Sternum orange with brown margins. Opisthosoma yellowish cream colored with same brown pattern as in male (Fig. 1B). Spinnerets as in male. Total length 4.1. Prosoma: 1.8 long, 1.8 wide. Opisthosoma: 2.1 long, 1.4 wide. Eyes: diameters: 0.12, 0.13, 0.10, 0.12; interdistances: 0.09, 014, 0.20, 0.20, 0.09, 0.06. Legs (2143): I: 5.0 (1.5, 0.8, 1.3, 1.0, 0.4); II: 5.6 (1.8, 0.9, 1.3, 1.2, 0.4); III: 4.9 (1.6, 0.8, 1.1, 1.0, 0.4); IV: 6.5 (1.8, 0.8, 1.8, 1.6, 0.5); femora I–III: p1-1-1, d0-1-1, r1-1-1; tibiae I–III: v2-2-2; tibia IV: p1-0-1; d0-0-1; r1-0-1; v1p-2-2; metatarsi I–II: v2-0-0; metatarsus III: p0-1-0; v2-0-0; metatarsus IV: p1-1-1; r1-1-1; v2-2-0. Female palps with single, pectinate claw, with three teeth (Fig. 2C); spination: femur p0-0-1; d0-1-2; tibia: p2-1, d1-0; r1-1; tarsus: p2-1; r1-1. Epigyne: divided into median septum and lateral lobes; epigynal field as long as wide; median septum small, as wide as long with posterior margin procurved; lateral lobes with large triangular projections (Figs 4D, 5D). Vulva: copulatory ducts long and coiled; blind ending appendage globose, adjacent to spermathecae, arising from copulatory duct; spermathecae globose; fertilization ducts laterad (Figs 4E–F, 5E).

##### Variation.

Males (n = 2): total length 4.2–4.4; prosoma length 2.0–2.1. Females (n = 4): total length 3.5–4.4; prosoma length 1.7–2.0; femur I length 1.4–1.6.

##### Distribution.

Known only from the type locality.

## Supplementary Material

XML Treatment for Diminutella

XML Treatment for Diminutella cortina
